# Association between chronic conditions, multimorbidity, and dependence levels in Chinese community-dwelling older adults with functional dependence: a cross-sectional study in south-central China

**DOI:** 10.3389/fpubh.2024.1419480

**Published:** 2024-09-20

**Authors:** Heng-Yu Hu, Ming-Yue Hu, Hui Feng, Pan-Pan Cui

**Affiliations:** ^1^Department of Nursing, Henan Provincial Key Medicine Laboratory of Nursing, Henan Provincial People’s Hospital, Zhengzhou University People’s Hospital, Zhengzhou, Henan, China; ^2^Xiang Ya Nursing School, Central South University, Changsha, Hunan, China

**Keywords:** chronic conditions, multimorbidity, functional dependence, older adults, community

## Abstract

**Background:**

The rising prevalence of multimorbidity and functional dependence in community-dwelling older adults contribute to the demand for home care services. Evidence on how chronic conditions, especially multimorbidity, affect dependence levels among older adults with functional dependence in a socio-cultural context is much needed to inform policy, workforce, aged care service development to meet the care needs of this population.

**Objectives:**

This study aimed to determine the association between chronic conditions, multimorbidity and dependence levels among Chinese community-dwelling older adults with functional dependence.

**Methods:**

A cross-sectional study was conducted with 1,235 community-dwelling older adults with functional dependence in Hunan province, China, from June to October 2018. Data on socio-demographic factors, cognitive function, vision and hearing conditions, activities of daily living (ADLs), and health conditions were collected, and binary logistic regression analyses were used to determine the association between chronic conditions, multimorbidity and dependence levels, with adjustments for relevant covariates.

**Results:**

Among the participants, 62.9% had multimorbidity. Parkinson’s disease, stroke, COPD, hypertension, mood and psychotic disorders (Anx/Sch/Dep) were significantly associated with high levels of functional dependence. After adjusting for demographic variables, cognitive function, vision, and hearing conditions, we observed a significant relationship between multimorbidity and higher functional dependence, but this association became insignificant when including certain chronic diseases closely associated with high-level dependence. Study revealed that Parkinson’s disease and stroke notably increase dependency risk across seven ADL domains, demonstrating their extensive impact on daily functioning.

**Conclusion:**

The prevalence of multimorbidity among Chinese community-dwelling older adults with functional dependence is very high. The association of multimorbidity with functional dependence is mediated by specific chronic conditions. These findings highlight the necessity of adopting an integrated care model that combines medical and social care, with a particular emphasis on managing multimorbidity and critical chronic conditions that lead to severe functional dependence to preventing and diminish the onset of disabilities.

## 1 Introduction

As of 2019, the population aged 65 and above constituted 11.5% of China’s total population, with projections indicating an increase to 16.9% by 2030 (1). Simultaneously, there has been a significant rise in the number of older adults in China requiring long-term care due to functional dependence. Functional dependence, defined as the need for assistance with activities of daily living (ADLs) such as bathing, dressing, and eating, further strains healthcare systems and caregivers (2). By the end of 2018, approximately 40 million older adults with functional dependence in China, and this figure is expected to surge to 97.5 million by 2050, thereby amplifying the demand for extensive and complex medical services (3). Based on the findings from the Seventh National Population Census in China, approximately 77.25% of older adults with functional dependence reside with their families, while a smaller fraction, around 8.28%, live in nursing institutions (4). The provision of ADLs is a crucial part of community aged-care services for helping older people to remain in their own homes for as long as possible. A person’s ability to perform ADLs is also widely used as an assessment tool to measure functional dependence in the application for entering government-subsidized home care services in China (5).

Providing adequate ADLs support for older adults is the first step in managing functional dependence, significantly enhancing their quality of life and reducing the burden on institutional care facilities (6, 7). However, further research is needed to identify key factors that help minimize or prevent progression to severe functional dependence. Research shows that individuals initially experiencing functional dependence are more likely to progress to more severe levels of dependence if not promptly intervened, resulting in escalating functional decline (8, 9). Increased severity of functional dependence correlates with a higher risk of mortality in older adults (10). Additionally, those with severe functional dependence are more susceptible to adverse health outcomes, such as higher healthcare utilization and reduced quality of life (11, 12). Evidence indicates that individuals who are initially disabled are more likely to undergo status changes and transition more rapidly through various stages than those without disabilities. This suggests that preventive actions and targeted welfare policies could help vulnerable populations avoid future health shocks and the progression to severe disability and death (13). Therefore, early and simple interventions during the initial stages of functional dependence can reduce the need for more intensive measures later for those at high risk of severe functional dependence (14).

Interventions to reduce dependence in older adults should focus not only on ADLs support but also on better management of disabling medical conditions before they progress to higher levels of disability. This is because various complex factors contribute to functional decline, with chronic health conditions being the primary contributors, particularly among older adults (14). The onset of chronic diseases can cause an initial decline in functional independence, progressing through mild, moderate, and severe dependence stages, ultimately leading to total disability (15). Crucially, research shows a strong link between multimorbidity in older adults and functional decline, exacerbating dependence and significantly impacting self-care abilities (16). Multimorbidity, defined as the presence of two or more chronic conditions (14), is notably more common among older adults. Studies indicate that multimorbidity prevalence is 66.1% in high-income countries (17) and 49.8% in low-and middle-income countries (18). Individuals with multimorbidity often experience higher healthcare costs and greater healthcare utilization (19), along with increased mortality rates (20) and a diminished quality of life (21). Furthermore, the elevated incidence of multimorbidity, coupled with functional dependence among community-dwelling older adults, significantly contributes to the increasing demand for home care services (22). Research suggests that multimorbidity significantly affects functional decline, irrespective of age, individual diseases, or geographical location (23, 24). This association is likely because the rapid accumulation of multiple chronic conditions in older adults disrupts the body’s homeostasis and reduces resilience to internal and external stressors. This disruption can lead to frailty (25), a medical syndrome that increases the risk of functional decline, dependence, and mortality (26).

Thus, examining the relationship between chronic conditions and multimorbidity with dependence levels in Chinese older adults requiring long-term care is crucial for developing targeted interventions. Effective early interventions can help maintain or improve functional status, potentially delaying the progression to severe functional dependence and reducing the associated healthcare burden. However, existing research predominantly focuses on the link between specific diseases, multimorbidity, and increased functional decline in the broader population of older adults, considering functional dependence as an isolated and dichotomous variable (27, 28). The impact of multimorbidity and chronic conditions on dependence levels among functionally dependent older adults remains under-explored. Notably, population-based studies in this field are lacking, especially in developing countries such as China, where long-term care (LTC) is underdeveloped (29). This study addresses this gap in research with an aim toward helping to inform policy, workforce, and LTC service development decision to meet the care needs of older adults in China and potentially other countries around the world, ultimately enhancing the quality of life and longevity of older adults with functional dependence.

Therefore, this study examines the relationship between chronic conditions, multimorbidity, and dependence levels among community-dwelling older adults with functional dependence in China. Initially, it assesses the prevalence of common chronic conditions and multimorbidity among adults aged 60 and above with functional dependence in Hunan province, south-central China. Subsequently, the study investigates the association between these chronic conditions, multimorbidity, and dependence levels in this demographic. Finally, it aims to elucidate how various health conditions contribute to dependency across different ADL domains.

## 2 Methods

### 2.1 Design

We conducted a population-based cross-sectional study in Hunan Province, south-central China, from June to October 2018.

### 2.2 Participants and settings

This study is part of a large project entitled *The Long-term Care Needs Assessment* (LCNA). Data were collected through a face-to-face questionnaire survey targeting older adults aged 60 and above who have functional dependence. The LCNA aimed to identify care and support needs among this demographic in south-central China, employing a multistage, cluster sampling method. In stage one, two cities or regions from all the 14 administrative regions of Hunan Province based on the National Bureau of Statistics of China were randomly selected (30). In stage two, two districts or counties were randomly chosen from each selected city or region. In the third stage, two sub-districts or townships were randomly selected from each district or county. Finally, according to the documented health records provided by local health centers, older adults aged 60 and over living with functional dependence were included with the assistance of the local Ministry of Civil Affairs.

This study was conducted according to the World Medical Association Declaration of Helsinki and approved by the Central South University Ethics Committee (Approval No. 2018011). Researchers provided participants with information about the study and gained their written informed consent before enrolling. The recruitment started in June, 2018 and was completed in October, 2018. Participants were included if they: (a) were aged 60 or over; (b) had lived in their own homes for at least 6 months within 1 year before the survey; (c) had difficulties or were unable to carry out ADLs according to the Barthel Index (BI) score (Barthel’s score < 100); (d) had signed the informed consent to participate in this study; and (e) had proxy respondents (e.g., family or primary caregivers) that had taken care of them for at least 1 month if they were unable to communicate.

**Figure 1:**
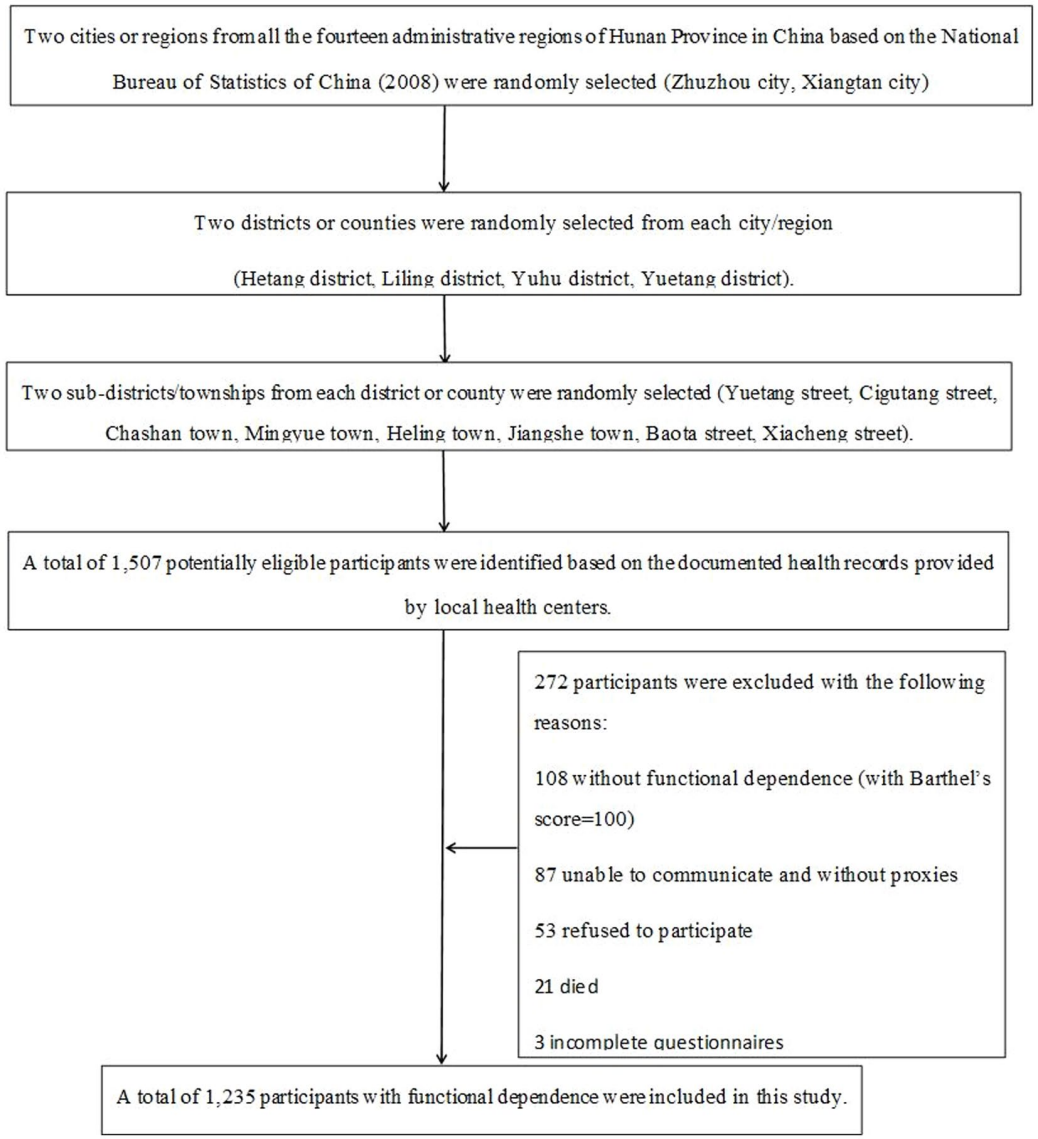
Sampling process flowchart.

In total, 1,507 potentially eligible participants were identified. After identifying potential participants, invitations were made via telephone by staff from the local health service centers. Subsequently, 53 of them were excluded for refusing to participate, 21 were excluded due to their death before the interview, and 87 were excluded due to their inability to communicate and lack of proxy respondents. In addition, 108 participants without functional dependence based on the Barthel Index (Barthel’s score = 100), and 3 participants with missing data were removed from the data analysis. Consequently, 1,235 participants with a Barthel’s score less than 100, indicating functional dependence, were included in the study. Figure 1 illustrates the selection process for the target population.

### 2.3 Measures

#### 2.3.1 Functional dependence

In this study, we employed the Barthel Index (BI) to assess individual levels of dependence in ADLs (31). The BI generates a cumulative score from 10 ADL domains, each evaluated using a weighted numerical scale. These domains fall into two categories: self-care (covering feeding, grooming, bathing, dressing, bowel and bladder control, toilet use) and mobility (including walking, transfers, stair climbing). Scores range from 0, indicating total dependence, to 100, signifying complete independence. Assessments typically take 5–10 min. Prior research has validated the BI’s reliability via face-to-face interviews (32). The Chinese version of the BI, widely used in older adult Chinese populations, classifies scores into four levels: 0–40 for complete dependence, 45–60 for severe dependence, 65–95 for slight dependence, and 100 for complete independence (33). In our research, the Cronbach’s alpha for this version was 0.91.

#### 2.3.2 Chronic conditions and multimorbidity

In our research, we identified 15 prevalent mental, cognitive, and physical health conditions: osteoarthritis, osteoporosis, stroke, dementia, Parkinson’s disease, coronary heart disease, hypertension, heart failure, chronic obstructive pulmonary disease (COPD), renal disease, depression, anxiety, schizophrenia, cancer, and diabetes. The selection of these conditions was based on a combination of literature review and expert opinions, with guidance from the “World Report on Ageing and Health” by the WHO, which identifies common causes of health-related disability in older adults (14), and the “China Country Assessment Report on Ageing and Health” by the WHO, which highlights major chronic conditions in the Chinese context (34). Multimorbidity was defined as the coexistence of two or more of these chronic conditions. Our research required that the chronic conditions be diagnosed by a doctor, thereby excluding conditions suspected by the older adults and family members but without diagnosis. Participants were firstly asked, “Have you ever been told by a doctor, nurse, or other health professional that you have these chronic conditions?” Then a table with a pre-defined list of 15 common chronic conditions was shown to the participants. If the answer to any condition was “yes,” further review of the medical records in the community health centers was required.

#### 2.3.3 Covariates

Sociodemographic variables encompassed age, gender (male, female), educational level (illiterate, primary, junior middle school or higher), marital status (married, single including never married, divorced, or widowed), presence of children (none, at least one child), living arrangements (alone, with others), and health insurance coverage (uninsured, insured). We assessed cognitive function in functionally dependent older adults using a tailored Cognitive Function Screening Questionnaire. This demographic often faces physical limitations, sensory impairments in vision and hearing, and challenges in expression and comprehension, which hinder the effectiveness of conventional cognitive tests such as the Mini-Mental State Examination (MMSE) and Clock Drawing Test. The questionnaire evaluates short-term memory, procedural memory, orientation, and decision-making ability on a scale of 0 to 40, with higher scores indicating poorer cognitive function. Scores are classified as: 0–1 for normal cognitive function, 2–11 for mild cognitive impairment, 12–25 for moderate cognitive impairment, and 26 or above for severe cognitive impairment. Validated in the Chinese older adults, the tool showed a Cronbach’s alpha of 0.893, split-half reliability of 0.913, test–retest reliability of 0.896, inter-rater reliability of 0.918, and a correlation with the MMSE of 0.914. Vision and hearing were assessed through self-reports or evaluator judgments, categorized as normal, mild, moderate, or severe impairment.

### 2.4 Statistical analysis

We calculated the median and interquartile range (IQR) for continuous variables and frequencies for categorical variables. To analyze the distribution and determinants of functional dependence in our study population, participants were categorized into two groups: the ‘Higher Dependence Group’ and the ‘Lower Dependence Group’. This classification was based on the median BI score, with those scoring below the median placed in the Higher Dependence Group (indicating a greater need for ADLs) and those scoring above the median placed in the Lower Dependence Group. Then binary logistic regression analyses were used to determine the association between chronic conditions, multimorbidity and the level of ADLs dependence, with adjustments for relevant covariates. Our regression analysis involved three progressively adjusted models to explore variable relationships. The first model examined the association between multimorbidity and higher functional dependence. The second model included all chronic conditions to investigate their correlation with greater functional dependence. In the third model, we assessed whether multimorbidity was significantly related to higher dependence independently of individual diseases by incorporating those diseases most strongly associated with increased dependence. Additionally, we employed binary logistic regression to analyze the association of chronic conditions and multimorbidity with dependence across 10 ADL domains, categorizing individuals in each domain as either ‘dependent’—those requiring any assistance—or ‘independent’.

All models were adjusted for sociodemographic variables, cognitive function, vision and hearing. Data analysis was conducted using SPSS version 25.0. Odds ratios (OR) with 95% confidence intervals (CI) were determined, with entry and removal criteria for variables set at 0.05 and 0.10, respectively. A *p*-value less than 0.05 was considered statistically significant. Multicollinearity testing showed that all variance inflation factors (VIF) for the independent variables were under 3, suggesting minimal correlation (35).

## 3 Results

### 3.1 Characteristics of participants

Table 1 indicates that the study included 1,235 participants. The Median (IQR) for age was 78 (71, 83), and the Median (IQR) for ADLs was 60 (35, 80). Based on the median ADLs score, 44.5% of participants exhibited lower functional dependence, while 55.5% showed higher functional dependence. Among the participants, 50.1% were female, 50.6% had completed primary school education, 58.2% were married, 95.7% lived with at least one child, 91.2% resided with others, and 97.5% had medical insurance. Regarding cognitive function, 32.1% of participants had normal function, 29.0% had mild cognitive impairment, 17.9% had moderate impairment, and 21.1% had severe impairment. For vision condition, 13.2% had normal vision, 57.2% mild impairment, 24.9% moderate impairment, and 4.8% severe impairment. Hearing condition was categorized as follows: normal function (29.5%), mild impairment (45.1%), moderate impairment (18.8%), and severe impairment (6.6%). Table 2 illustrates the prevalence of common chronic conditions and multimorbidity, revealing that 86.9% had at least one chronic disease and 62.9% experienced multimorbidity.

**Table 1:** Participants’ characteristic and functional status of the study population (*N* = 1,235).

Characteristics	Total, *n* (%)
Age, y	
Median [IQR]	78 [71,83]
Gender, no. (%)	
Male	616 (49.9)
Female	619 (50.1)
Education, no. (%)	
Illiterate	225 (18.2)
Primary school	625 (50.6)
Middle school and above	385 (31.2)
Marital status, no. (%)	
Married	719 (58.2)
Single (never married, divorced, or widowed)	516 (41.8)
Child status, no. (%)	
No children	53 (4.3)
With at least one child	1,182 (95.7)
Living condition, no. (%)	
Living alone	109 (8.8)
Living with others	1,126 (91.2)
Medical insurance, no. (%)	
Uninsured	31 (2.5)
Insured	1,204 (97.5)
Cognitive function	
Normal	396 (32.1)
Mild impairment	358 (29.0)
Moderate impairment	221 (17.9)
Severe impairment	260 (21.1)
Vision condition	
Normal	163(13.2)
Mild impairment	706(57.2)
Moderate impairment	307 (24.9)
Severe impairment	59 (4.8)
Hearing condition	
Normal	364 (29.5)
Mild impairment	557 (45.1)
Moderate impairment	232 (18.8)
Severe impairment	82 (6.6)
Number of chronic conditions, no. (%)	
0	161 (13.0)
1	297 (24.0)
2	325 (26.3)
3	231 (18.7)
4	126 (10.2)
5	61 (4.9)
≥6	34 (2.8)
Multimorbidity	777 (62.9)
Functional status (ADLs)	
Median [IQR]	60 [35,80]
Lower dependence	549 (44.5)
Higher dependence	686 (55.5)
Dependence in 10 domains of ADLs, no. (%)	
Stair climbing	1,121 (90.8)
Bathing	939 (76.0)
Walking	865 (70.0)
Bladder	845 (68.4)
Dressing	818 (66.2)
Toilet use	806 (65.3)
Transfers	795 (64.4)
Bowels	698 (56.5)
Grooming	585 (47.4)
Feeding	552 (44.7)

IQR, interquartile range; ADLs, activities of daily living.

**Table 2:** Prevalence of common chronic conditions and multimorbidity (*N* = 1,235).

Chronic conditions	Total, *n* (%)
Hypertension	698 (56.5)
Coronary heart disease	493 (39.9)
Stroke	460 (37.2)
Diabetes	217 (17.6)
Osteoarthritis	164 (13.3)
Dementia	161 (13.0)
Osteoporosis	155 (12.6)
Heart failure	90 (7.3)
Chronic obstructive pulmonary diseases	78 (6.3)
Parkinson’s disease	48 (3.9)
Kidney disease	48 (3.9)
Cancer	31 (2.5)
Schizophrenia	20 (1.6)
Depression	7 (0.6)
Anxiety	3 (0.2)
Number of chronic conditions, no. (%)	
0	161 (13.0)
1	297 (24.0)
2	325 (26.3)
3	231 (18.7)
4	126 (10.2)
5	61 (4.9)
≥6	34 (2.8)
Multimorbidity	777 (62.9)

### 3.2 Associations between chronic conditions, multimorbidity, and higher dependence

To examined the relationships between chronic conditions, multimorbidity, and higher functional dependence, three models were calculated. Due to the low prevalence rates of anxiety, schizophrenia, and depression, these conditions were consolidated into a single independent variable into the regression analysis, denoted as Anx/Sch/Dep, to represent mood and psychotic disorders. The first model indicated a significant association between multimorbidity and higher functional dependence (OR = 2.194, 95% CI: 1.662–2.897), controlling for covariates. In the second model, five chronic conditions—stroke (OR = 2.744, 95% CI: 2.020–3.726), Parkinson’s disease (OR = 2.867, 95% CI: 1.358–6.052), hypertension (OR = 1.363, 95% CI: 1.007–1.845), COPD (OR = 2.034, 95% CI: 1.1.41–3.626), and Anx/Sch/Dep (OR = 4.278, 95% CI: 1.362–13.436)—were found to be significantly associated with higher dependence. The third model revealed that after adjusting for diseases significantly associated with high-level dependence, multimorbidity was not significantly associated with higher functional dependence. Table 3 displays the significant results of the model in relation to the dependent variables. Detailed information on the assignment of independent variables in multivariate binary logistic regression analysis, along with comprehensive results of three models examining the relationship between chronic disease, multimorbidity, and higher dependence, is provided in the Supplementary material.

**Table 3:** Multivariate binary logistic regression analysis of factors associated with higher functional dependence.

Variables	Model 1^a^				Model 2^b^				Model 3^c^			
	*P*-value	OR	95%CI		*P*-value	OR	95%CI		*P*-value	OR	95%CI	
Multimorbidity	<0.001***	2.194	1.662	2.897					0.127	1.350	0.918	1.984
Osteoarthritis					0.052	1.542	0.997	2.385				
Osteoporosis					0.075	1.470	0.962	2.248				
Stroke					<0.001***	2.744	2.020	3.726	<0.001***	2.494	1.826	3.407
Dementia					0.180	1.430	0.848	2.413				
Parkinson’s disease					0.006**	2.867	1.358	6.052	0.014*	2.524	1.207	5.278
Coronary heart disease					0.457	0.892	0.659	1.206				
Hypertension					0.045*	1.363	1.007	1.845	0.366	1.181	0.824	1.692
Heart failure					0.095	1.595	0.922	2.758				
COPD					0.016*	2.034	1.141	3.626	0.011*	2.090	1.185	3.686
Kidney disease					0.093	1.880	0.900	3.928				
Anx/Sch/Dep					0.013*	4.278	1.362	13.436	0.010*	4.424	1.424	13.741
Cancer					0.531	1.299	0.573	2.943				
Diabetes					0.599	1.104	0.764	1.594				

^a^Model 1 included multimorbidity and Covariates; ^b^Model 2 included 15 chronic conditions and Covariates, where anxiety, schizophrenia, and depression were consolidated into a single independent variable, denoted as Anx/Sch/Dep; ^c^Model 3 included the variables for multimorbidity and the diseases significantly associated with higher functional dependence in model 2; *p < 0.05, **P < 0.01, ***P < 0.005.

### 3.3 Factors associated with dependence in 10 ADL domains

Utilizing binary logistic regression analysis, our study further investigates the correlation between chronic conditions, multimorbidity, and dependency across various ADL domains. The analysis specifically identifies diseases that are significantly associated with increased functional dependency. Notably, Parkinson’s disease and stroke were linked to a substantially higher risk of care dependency in seven ADL domains, including feeding, bathing, grooming, dressing, toilet use, transferring, and walking. COPD was significantly associated with dependency in five ADL domains: bathing, grooming, dressing, toilet use, and transferring. The combined conditions of anxiety, schizophrenia, and depression (Anx/Sch/Dep) showed strong correlations (OR > 4) in the domains of feeding, grooming, and dressing. Furthermore, hypertension was significantly related to an increased risk of uncontrolled bowel movements and urination. These findings are elaborated in Table 4.

**Table 4:** Multivariate binary logistic regression analysis of factors associated with dependence in 10 domains of ADLs.

Domains	Variables	OR	95%CI		*p*-value
Feeding	Parkinson disease	4.488	2.194	9.181	<0.001***
	Anx/Sch/Dep	4.396	1.536	12.579	0.006**
	Stroke	2.547	1.850	3.508	<0.001***
	Dementia	1.690	1.047	2.728	0.032*
	Osteoarthritis	1.643	1.076	2.509	0.021*
Bathing	Stroke	2.749	1.851	4.082	<0.001***
	Parkinson disease	2.658	1.037	6.812	0.042*
	Dementia	2.611	1.166	5.847	0.020*
	COPD	2.379	1.164	4.863	0.017*
	Heart failure	2.196	1.107	4.355	0.024*
Grooming	Anx/Sch/Dep	4.641	1.624	13.265	0.004**
	Parkinson disease	3.549	1.743	7.228	<0.001***
	COPD	3.159	1.789	5.576	<0.001***
	Stroke	2.687	1.958	3.687	<0.001***
	Heart failure	1.820	1.069	3.096	0.027*
Dressing	Anx/Sch/Dep	4.260	1.104	16.435	0.035*
	Parkinson disease	3.894	1.599	9.482	0.003**
	Stroke	2.944	2.066	4.195	<0.001***
	COPD	2.074	1.106	3.888	0.023*
	Osteoarthritis	1.745	1.077	2.827	0.024*
Bowels	Hypertension	1.679	1.188	2.372	0.003**
	Diabetes	1.545	1.077	2.217	0.018*
Bladder	Osteoporosis	1.636	1.040	2.572	0.033*
	Hypertension	1.482	1.035	2.122	0.032*
Toilet use	Dementia	2.465	1.285	4.728	0.007**
	Parkinson disease	2.262	1.027	4.982	0.043*
	Stroke	2.026	1.447	2.839	<0.001***
Transfers	Stroke	2.682	1.927	3.732	<0.001***
	Parkinson disease	2.483	1.147	5.375	0.021*
	COPD	2.004	1.106	3.633	0.022*
	Osteoporosis	1.769	1.135	2.757	0.012*
Walking	Stroke	1.580	1.129	2.211	0.008**
	Parkinson disease	2.702	1.122	6.503	0.027*

All models were adjusted for sociodemographic variables, cognitive function, vision and hearing; *P < 0.05, **P < 0.01, ***P < 0.005.

## 4 Discussion

Our study determined the prevalence of common chronic conditions and multimorbidity among older adults living with functional dependence in Hunan province, south-central China. Among the 1,235 participants, 1,074 (86.9%) had at least one chronic disease, and 777 (62.9%) experienced multimorbidity. This finding is notably higher compared to studies focusing on the general aging population in China, which reported multimorbidity prevalences ranging from 43.6 to 47.5% (27, 28). This difference suggests that older adults living in the community with functional dependence might have unique health profiles and care needs that are not fully captured in broader population studies. This implies that health care strategies and policies need to be tailored to address the specific challenges faced by this demographic. Similarly, a study conducted in Shanghai, China, focusing on community-dwelling older adults with physical disabilities, reported a higher prevalence of multimorbidity, with 74.3% of individuals affected (36). The increased prevalence observed in the Shanghai study can be attributed to the study exclusively involving older adults with severe physical disabilities, suggesting a potential correlation between the severity of functional impairment and the prevalence of multimorbidity in the older adults.

The increasing prevalence of multimorbidity among individuals with functional dependence highlights the urgent need for integrated care models. Previous research indicates that Chinese older adults with multimorbidity and ADLs dependence tend to utilize more health care services, including frequent transitions between hospital and home care settings, and have higher unmet care needs (22). A similar trend is observed in the United States, where heart failure patients with multimorbidity and ADLs limitation experience elevated mortality rates and increased health care utilization (37). Consequently, there is an urgent need for robust integrated care strategies that integrate medical and social care for Chinese older people, especially those with multimorbidity and functional dependence. Such approaches should consider not only the medical management of multiple chronic conditions but also the support needed for daily functioning and quality of life (38). Findings from our population-based study highlight the importance of policy and resource development to support integrated care models, ensuring that the unique needs of this growing demographic are adequately met.

Our study finds that Parkinson’s disease, stroke, COPD, hypertension, mood and psychotic disorders (Anx/Sch/Dep) are significantly correlated with higher functional dependence in older adults living in Chinese communities. Notably, Parkinson’s disease contributes more to increased dependence, indicating a more severe impact compared to other conditions. Consistent with other studies, Parkinson’s disease is linked to severe disability (39) and represents the condition with the highest risk for long-term care dependency (24). The significant risk for severe disability and long-term care dependency in Parkinson’s disease is primarily due to its progressive motor symptoms, such as tremors, rigidity, and bradykinesia, which severely impair daily living activities. Additionally, non-motor symptoms like cognitive impairment and mood disorders further exacerbate disability, creating a complex clinical condition that accelerates functional decline and increases reliance on caregivers (40, 41). Stroke is also a major contributor to severe functional dependence in our study. Similarly, the Global Burden of Disease project highlights stroke as the leading cause of disability in China’s population aged 60 and above (42). In the Belgian population, stroke is also a major contributor to severe disability among the older adults (43). Evidence from the Global Burden of Disease (GBD) study shows that stroke is the second leading cause of death and the third leading cause of disability worldwide (44). The GBD predicts that the burden of stroke among older adults will increase by 44% between 2004 and 2030 (45). Additionally, 89% of this burden is concentrated in developing countries (46). In China, the lifetime risk of stroke is 39.9%, the highest globally (47). Recently, China has recorded nearly 2.5 million new stroke cases annually, with 75% of survivors suffering from persistent functional impairments (48). Therefore, improved stroke management in developing countries, especially during the initial stages of functional impairment, is essential to prevent severe disability, reduce mortality, and decrease the need for long-term institutional care. Previous studies report that COPD is associated with a reduced ability to perform basic self-care and independent living tasks, showing greater disability than other chronic health conditions (49–51). Our findings corroborate this, indicating a significant correlation between COPD and increased dependence. The association of hypertension with severe disability was also observed in our study. Hypertension, a significant contributor to cardiovascular disease, declines in intrinsic capacity, and premature death, warrants particular attention (52). Contrary to other research, dementia did not emerge as a major contributor to severe disability in our study (53). A plausible explanation is that cognitive function, highly correlated with dementia and included as a confounder in our analysis, may be a broader and more significant indicator for ADL decline than dementia alone.

Notably, mood and psychotic disorders (Anx/Sch/Dep) show a strong correlation with significant functional dependence in our study. The disability resulting from mental illness, which often manifests in cognitive, affective, and behavioral disorders, can severely limit daily activities and social participation (54). However, our research finds a notably low detection rate of anxiety and depression, consistent with findings from other studies indicating that the detection rate of mental disorders among older individuals within Chinese primary care settings is considerably low (55). The cultural stigma associated with mental health and the scarcity of mental health professionals in community settings may contribute to the potential underestimation of anxiety and depression (56). Furthermore, the recognition rate of schizophrenia surpasses that of anxiety and depression in our study, likely due to the community mental health system in China prioritizing severe mental disorders amid limited resources and political concerns for social safety, particularly focusing on patients exhibiting violent or socially disruptive behaviors (57). While our findings are promising, considering these limitations, our results should be interpreted with caution, especially because the specific conditions contributing to high functional dependence are not definitively identified, and our categorization of mood and psychotic disorders only includes three types. Future research is needed to explore methods for more accurately detecting mental health conditions among the community-dwelling older population, employing different data collection methods to validate our findings, thereby enriching our understanding of each condition’s impact on functional dependence and enhancing the development of targeted interventions in primary care and mental health services.

Our study reaffirms the significant associations previously reported for Parkinson’s disease, stroke, COPD, hypertension, mood and psychotic disorders (Anx/Sch/Dep) with high levels of functional dependence, not only in the general older adult population but also among those requiring long-term care. Therefore, addressing chronic conditions closely linked to functional decline is crucial. These conditions pose complex, long-term challenges for caregivers, and a comprehensive response is necessary to manage major chronic conditions effectively. This approach aims to minimize the risk of severe functional dependence, prevent the accumulation of functional deficits, reduce hospitalization and high-cost interventions, and decrease premature deaths in at-risk populations.

Our findings revealed a significant association between multimorbidity and high levels of functional dependency after adjusting for demographic variables, cognitive function, and sensory abilities. However, this association ceased to be significant upon the inclusion of certain chronic diseases that are strongly linked to high-level dependency. This suggests that while multimorbidity is initially a predictor of greater functional dependence, its impact may be mediated by the presence of specific chronic conditions. This finding underscores the importance of identifying and managing key chronic diseases that contribute significantly to functional decline. Furthermore, our study highlights the necessity of a comprehensive approach in assessing the functional status of the older adults, considering not only the count of chronic conditions but also their nature and severity. While multimorbidity is an important factor to consider in the context of functional dependence, the role of specific chronic diseases in driving this dependence cannot be overlooked.

While existing research has acknowledged a connection between multimorbidity and increased functional decline, a clear distinction between the effects of multimorbidity and single diseases remains underexplored (58). The existing body of literature reveals inconsistent results: for instance, a German cohort study identified a correlation between multimorbidity and care dependence, controlling for other associated chronic conditions (24). Conversely, a UK study incorporating both common chronic conditions and multimorbidity into its multivariate analysis found no significant correlation between the number of conditions and the decline in functional status (59). The variability in multimorbidity measurements, the diversity of health conditions, and the specific characteristics of study participants complicate international comparisons among different studies. Consequently, future research should address these challenges, highlighting the need for a standardized definition and classification system for multimorbidity. Furthermore, while most studies focus on the relationship between the type and number of chronic conditions and functional decline, factors such as disease severity, duration, and combinations of chronic diseases might play a more significant role in exacerbating multimorbidity. Therefore, it is essential to conduct additional research that examines the severity, duration, and patterns of multimorbidity to deepen our understanding of these relationships.

Our study delved into the relationship between chronic conditions, multimorbidity, and dependency across various ADL domains. It notably found that Parkinson’s disease and stroke significantly elevate the risk of dependency in seven ADL domains, underscoring their extensive impact on daily functioning. These insights necessitate the customization of nursing care services, as well as education and training, to effectively manage these diseases. Focused nursing care and specific education and training programs are crucial to mitigate the activities impacted by Parkinson’s disease and stroke, aiming to delay the onset of disability and improve patient autonomy. Furthermore, the combined conditions of anxiety, schizophrenia, and depression (Anx/Sch/Dep) exhibited strong correlations in activities like feeding, grooming, and dressing. This correlation may stem from the social withdrawal and reduced interest in personal appearance and hygiene commonly seen in individuals with these mental health issues. This finding highlights the profound influence of mental health on functional abilities and underscores the need to integrate mental health care into the treatment plans for older adults experiencing these conditions.

This study presents several limitations. Firstly, our analysis does not cover all chronic conditions. While we identified multimorbidity using 15 chronic conditions, we included conditions like anxiety are less prevalent. This indicates a need for refining the list of conditions that are pertinent to functional decline. Secondly, as a cross-sectional study, it cannot establish causal relationships, hence the results should be interpreted as correlations with caution. Thirdly, our study relies on self-reports and medical record reviews for assessing chronic conditions and multimorbidity. Given the context of primary care settings in Chinese communities, this approach may lead to low detection rates of mental disorders such as depression and anxiety, potentially compromising the accuracy of our findings. Lastly, this research, which for the first time examines the relationship between chronic conditions, multimorbidity, and functional status in older adults with functional dependence, is restricted to a single Chinese province. Future research should aim to involve larger, nationally representative samples to broaden the understanding of these associations and to validate our findings in various regions and demographic groups across China.

## 5 Conclusion

Our research indicates a notably high prevalence of multimorbidity among Chinese community-dwelling older adults with functional dependence. We observe the significant associations for Parkinson’s disease, stroke, COPD, hypertension, mood and psychotic disorders (Anx/Sch/Dep) with high levels of functional dependence. Furthermore, our findings suggest the association of multimorbidity with functional dependence is mediated by specific chronic conditions, diminishing its significance as an independent predictor when these conditions are accounted for. These results emphasize the necessity of implementing an integrated care model for the community-dwelling older adults in China, especially for those experiencing multimorbidity and functional dependence. This model should comprehensively include both medical and social care services, with a particular emphasis on managing critical chronic conditions that contribute to severe functional dependence. In addition, policy makers should especially target those activities significantly affected by primary chronic conditions such as Parkinson’s disease and stroke when designing community-based long-term care services. This could potentially contribute to supporting disabled and older adults in community-living situations. Further research is needed to conclusively determine the extent to which such services can delay disability onset and optimize functional autonomy. Furthermore, in assessing and managing the risk of significant functional dependence among older adults in the community with prevalent multimorbidity, merely concentrating on the quantity of concurrent diseases is inadequate. It is essential to consider factors like the combination of comorbidities, and the duration and severity of specific diseases. Future research should investigate these elements more comprehensively to improve our understanding and approach to managing functional dependence risks in this population.

## Data Availability

The original contributions presented in the study are included in the article/Supplementary material, further inquiries can be directed to the corresponding author.
