# Supplementary material for: Association between chronic conditions, multimorbidity, and dependence levels in Chinese community-dwelling older adults with functional dependence: a cross-sectional study in south-central China
Source: Front Public Health. 2024 Sep 20;12:1419480. doi: 10.3389/fpubh.2024.1419480 (PMC11451049; doi:10.3389/fpubh.2024.1419480)
Supplement: Supplementary file 1 [file Data_Sheet_1.docx]

**Title:** Association between Chronic Conditions, Multimorbidity and Dependence Levels in Chinese Community-Dwelling older Adults with Functional Dependence: a cross-sectional study in south-central China

**Author information**

Heng-Yu Hu^1^, Ming-Yue Hu^2^, Hui Feng^2^, Pan-Pan Cui ^1*^

1 Department of Nursing, Henan Provincial Key Medicine Laboratory of Nursing, Henan Provincial People’s Hospital; Zhengzhou University People’s Hospital, Zhengzhou, Henan, China.

2 Xiang Ya Nursing School, Central South University, Changsha, Hunan, China

**The Supplementary Information List**

**Table 1** Multiple regression analysis of independent variable assignment

**Table 2** Multivariate binary logistic regression analysis of factors associated with higher functional dependence

**Table 1** Multiple regression analysis of independent variable assignment

| **Independent Variables** | **Independent Variable Assignment Methods** |
| --- | --- |
| Age | continuous variable |
| Gender | Male = 0; Female = 1 |
| Education | Illiterate=0; Primary school=1;  Junior middle school and above=2 |
| Marital status | Married=0; Single=1 |
| Living condition | Living alone=0; Living with others=1 |
| Child status | No children=0; With at least one child=1 |
| Medical insurance | Uninsured=0; insured=1 |
| Cognitive function | continuous variable |
| Vision condition | Normal=0; Mild impairment=1  Moderate impairment=2; Severe impairment=3 |
| Hearing condition | Normal=0; Mild impairment=1  Moderate impairment=2; Severe impairment=3 |
| Chronic conditions^a)^ | Without=0; With =1 |
| Multimorbidity | Without =0; With=1 |

*Notes.* a) i.e., with dementia vs. without dementia, including 15 common chronic conditions.

**Table 2** Multivariate binary logistic regression analysis of factors associated with higher functional dependence

|  | **Model 1^a)^** | | | | **Model 2^b)^** | | | | **Model 3^c)^** | | | |
| --- | --- | --- | --- | --- | --- | --- | --- | --- | --- | --- | --- | --- |
| **Variables** | P-value | OR | 95%CI | | P-value | OR | 95%CI | | P-value | OR | 95%CI | |
| Multimorbidity (ref = no) | 0.＜0.001*** | 2.194 | 1.662 | 2.897 |  |  |  |  | .127 | 1.350 | 0.918 | 1.984 |
| Demographic |  |  |  |  |  |  |  |  |  |  |  |  |
| Age (ref= young-old) |  |  |  |  |  |  |  |  |  |  |  |  |
| Old-old | 0.451 | 1.119 | 0.835 | 1.501 | 0.204 | 1.220 | 0.898 | 1.657 | .199 | 1.219 | 0.901 | 1.650 |
| Oldest-old | 0.965 | 1.013 | 0.573 | 1.790 | 0.286 | 1.379 | 0.764 | 2.488 | .304 | 1.357 | 0.759 | 2.427 |
| Gender (ref = male) | 0.048* | 0.752 | 0.567 | .997 | 0.209 | 0.826 | 0.613 | 1.113 | .214 | 0.831 | 0.620 | 1.113 |
| Medical insurance (ref= uninsured) | 0.800 | 0.892 | 0.370 | 2.153 | 0.889 | 1.068 | 0.424 | 2.690 | .971 | 1.017 | 0.412 | 2.508 |
| Education (ref= illiterate) |  |  |  |  |  |  |  |  |  |  |  |  |
| Primary school | 0.554 | 1.117 | 0.775 | 1.610 | 0.760 | 1.061 | 0.726 | 1.552 | 1.058 | 0.727 | 1.538 | 1.058 |
| Junior middle school and above | 0.018* | 1.658 | 1.092 | 2.518 | 0.060 | 1.522 | 0.983 | 2.358 | 1.561 | 1.017 | 2.397 | 1.561 |
| Marital status (ref= married) | 0.181 | 0.807 | 0.590 | 1.105 | 0.237 | 0.822 | 0.594 | 1.137 | 0.830 | 0.603 | 1.143 | 0.830 |
| Living condition (ref= Living alone) | 0.146 | 1.436 | 0.882 | 2.339 | 0.277 | 1.327 | 0.797 | 2.208 | 1.356 | 0.821 | 2.239 | 1.356 |
| Child status (ref= No children) | 0.496 | 0.785 | 0.392 | 1.575 | 0.334 | 0.703 | 0.344 | 1.437 | 0.699 | 0.345 | 1.415 | 0.699 |
| Cognitive Function (ref= Normal) |  |  |  |  |  |  |  |  |  |  |  |  |
| Mild impairment | ＜0.001*** | 2.294 | 1.670 | 3.152 | ＜0.001*** | 2.321 | 1.668 | 3.228 | ＜0.001*** | 2.282 | 1.647 | 3.161 |
| Moderate impairment | ＜0.001*** | 4.623 | 3.134 | 6.821 | ＜0.001*** | 4.437 | 2.954 | 6.665 | ＜0.001*** | 4.441 | 2.978 | 6.624 |
| Severe impairment | ＜0.001*** | 17.336 | 10.701 | 28.084 | ＜0.001*** | 16.581 | 9.751 | 28.195 | ＜0.001*** | 17.402 | 10.649 | 28.440 |
| Vision Condition (ref= Normal) |  |  |  |  |  |  |  |  |  |  |  |  |
| Mild impairment | 0.082 | 1.782 | 0.928 | 3.418 | 0.156 | 0.132 | 1.680 | 0.855 | 1.801 | 0.915 | 3.545 | 1.801 |
| Moderate impairment | 0.110 | 1.670 | 0.890 | 3.133 | 0.261 | 0.233 | 1.490 | 0.773 | 1.555 | 0.806 | 3.000 | 1.555 |
| Severe impairment | 0.327 | 0.698 | 0.339 | 1.434 | 0.159 | 0.167 | 0.588 | 0.277 | 0.616 | 0.291 | 1.306 | 0.616 |
| Hearing Condition (ref= Normal) |  |  |  |  |  |  |  |  |  |  |  |  |
| Mild impairment | 0.624 | 1.165 | 0.633 | 2.144 | 0.715 | 0.745 | 1.111 | 0.590 | 0.702 | 1.130 | 0.604 | 2.116 |
| Moderate impairment | 0.272 | 1.387 | 0.774 | 2.485 | 0.413 | 0.428 | 1.279 | 0.696 | 0.379 | 1.311 | 0.717 | 2.398 |
| Severe impairment | 0.357 | 0.749 | 0.405 | 1.385 | 0.505 | 0.495 | 0.800 | 0.422 | 0.428 | 0.773 | 0.409 | 1.461 |
| Chronic conditions (ref = no) |  |  |  |  |  |  |  |  |  |  |  |  |
| Osteoarthritis |  |  |  |  | 0.052 | 1.542 | 0.997 | 2.385 |  |  |  |  |
| Osteoporosis |  |  |  |  | 0.075 | 1.470 | 0.962 | 2.248 |  |  |  |  |
| Stroke |  |  |  |  | ＜0.001*** | 2.744 | 2.020 | 3.726 | ＜0.001*** | 2.494 | 1.826 | 3.407 |
| Dementia |  |  |  |  | 0.180 | 1.430 | 0.848 | 2.413 |  |  |  |  |
| Parkinson’s disease |  |  |  |  | 0.006** | 2.867 | 1.358 | 6.052 | 0.014* | 2.524 | 1.207 | 5.278 |
| Coronary heart disease |  |  |  |  | 0.457 | 0.892 | 0.659 | 1.206 |  |  |  |  |
| Hypertension |  |  |  |  | 0.045* | 1.363 | 1.007 | 1.845 | 0.366 | 1.181 | 0.824 | 1.692 |
| Heart failure |  |  |  |  | 0.095 | 1.595 | 0.922 | 2.758 |  |  |  |  |
| Chronic obstructive pulmonary diseases |  |  |  |  | 0.016* | 2.034 | 1.141 | 3.626 | 0.011* | 2.090 | 1.185 | 3.686 |
| Kidney disease |  |  |  |  | 0.093 | 1.880 | 0.900 | 3.928 |  |  |  |  |
| Mental health disorder |  |  |  |  | 0.013* | 4.278 | 1.362 | 13.436 | 0.010* | 4.424 | 1.424 | 13.741 |
| Cancer |  |  |  |  | 0.531 | 1.299 | 0.573 | 2.943 |  |  |  |  |
| Diabetes |  |  |  |  | 0.599 | 1.104 | 0.764 | 1.594 |  |  |  |  |

*Notes.* a) Model 1 included multimorbidity and Covariates; b) Model 2 included 15 chronic conditions and Covariates, where anxiety, schizophrenia, and depression were consolidated into a single independent variable, denoted as Anx/Sch/Dep; c) Model 3 included the variables for multimorbidity and the diseases significantly associated with higher functional dependence in model 2; **P* < 0·05, ***P* < 0·01, ****P* < 0·005
